# Case Report: Hexachloroethane Smoke Inhalation: A Rare Cause of Severe Hepatic Injuries

**DOI:** 10.1289/ehp.8635

**Published:** 2006-01-24

**Authors:** Ching-Hui Loh, Yaw-Wen Chang, Saou-Hsing Liou, Jun-Hei Chang, Hong-I Chen

**Affiliations:** 1 Department of Family Medicine and Community Health, Tri-Service General Hospital, National Defense Medical Center, Taipei, Taiwan, ROC; 2 Department of Public Health, National Defense Medical Center, Neihu, Taipei, Taiwan, ROC; 3 Division of Environmental Health and Occupational Medicine, National Health Research Institutes, Kaohsiung, Taiwan, ROC; 4 Department of Surgery, Tri-Service General Hospital, National Defense Medical Center, Taipei, Taiwan, ROC; 5 Medical Bureau, Department of National Defense, Taipei, Taiwan, ROC

**Keywords:** hepatotoxicity, hexachloroethane, white smoke, zinc oxide

## Abstract

**Context:**

We report on two patients, a 23-year-old man and a 24-year-old man, who had chemical pneumonitis and respiratory distress after inhaling hexachloroethane/zinc oxide (HC/ZnO) smoke during military training.

**Case Presentation:**

The patients had been healthy previously and denied any history of alcohol or drug abuse. Hematologic tests revealed leukocytosis with neutrophils predominant. The respiratory conditions of both patients improved after steroid therapy and oxygen support, but deterioration of liver function was found. The laboratory results showed that alanine aminotransferase (ALT) and γ-glutamyl transpeptidase levels were elevated about 1.5-fold the normal limits and that aspartate aminotransferase (AST) levels were marginally elevated. The elevation of liver aminotransferase started from day 1 and day 2 and peaked from day 18 to day 22. ALT/AST levels then returned to normal in 6 weeks. Common viral hepatitis was ruled out after serologic tests. Abdominal sonography and physical examination failed to show any specific findings.

**Discussion:**

The hepatotoxic effect was attributed to inhalation of high-concentration HC/ZnO smoke in an enclosed area, where several hepatotoxicants, including ZnCl_2_, HC, and chlorinated vapors, could have been generated and mixed in the smoke.

**Relevance to clinical practice:**

These case reports elaborate the hepatic effects that may occur in addition to pulmonary effects of HC/ZnO smoke.

Hexachloroethane/zinc oxide (HC/ZnO) smoke, also known as white smoke, has many military and civilian applications, such as in training exercises and on the battlefield [Agency for Toxic Substances and Disease Registry (ATSDR) 1997; Holmes 1999; Zerahn et al. 1999]. Inhalation is the most common route of injury. Documented injuries are predominately pulmonary and range from cough and dyspnea to chemical pneumonitis, pulmonary edema, adult respiratory distress syndrome, and death (Cullumbine 1957; Greenfield et al. 2002; Hjortso et al. 1988). The toxicity of HC/ZnO smoke results from both the chemical and physical properties of the smoke. The primary component of white smoke is HC/ZnO combined with granular aluminum. Upon burning, several compounds are produced: zinc chloride, 62.5%; ZnO, 9.6%; iron oxide, 10.7%; aluminum oxide, 5.4%; lead oxide, 1%; and chlorinated vapors, 10.8% (DeVaull et al. 1989). The reaction equation is as follows (Cichowicz 1983):


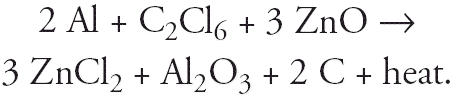


The reaction is exothermic and self-perpetuating to the right, liberating large amounts of ZnCl_2_ as a hot vapor. Upon cooling, it nucleates to form an aerosol that rapidly absorbs water from the surrounding atmosphere. Hydrated ZnCl_2_ particles then scatter light, thereby creating the desired obscurant effect (ATSDR 1997; Holmes 1999; Katz et al. 1980). When a smoke grenade is used, the heat causes other chemicals to form, including carbon tetrachloride, tetrachlorethylene, hexachlorobenzene, and phosgene (ATSDR 1997). Medical personnel, military and civilian alike, may be called upon to recognize and treat patients with HC/ZnO smoke injuries. Almost all studies have focused on the pulmonary effects, and little if any information exists on hepatic toxicity. In this article we report two cases of acute hepatic injuries associated with HC/ZnO smoke inhalation.

## Case Reports

### Patient 1.

The first patient, a 23-year-old man with an unremarkable medical and surgical history, was admitted to our hospital because of accidental inhalation of HC/ZnO smoke during military training. The patient had been healthy previously and denied any history of alcohol or drug abuse. This soldier specifically denied any history of smoking or pulmonary disorders. He had been in a tunnel without respiratory protection when a smoke grenade used to simulate battle conditions ignited near the entrance. The tunnel was about 1.75 m × 0.6 m × 360 m (5.74 ft × 1.97 ft × 1,181 ft). The estimated time of exposure to the HC/ZnO smoke was about 3 min. He suffered from lacrimation, cough, chest tightness, dyspnea, and sore throat at the scene.

On arrival at the hospital, the initial physical examination showed that the patient’s blood pressure was 110/70 mmHg, his heart rate was 80 bpm, and his oral temperature was 38.1°C (100.6°F). His respirations were shallow but unlabored, with a rate of 19/min. Auscultation of the lungs was unremarkable. The cardiac examination revealed a normal S_1_ and S_2_ without murmur, gallop, rub, or click. Arterial blood gas analysis and blood chemistry test values were within the normal ranges except that the alanine aminotransferase (ALT) and γ-glutamyl transpeptidase (GGT) levels were elevated about 1.5-fold of normal limits and the aspartate aminotransferase (AST) level was marginally elevated. Leukocytosis with neutrophils predominating was also noted (Table 1). The leukocytosis returned to normal on the 10th day after the event.

**Figure 1:**
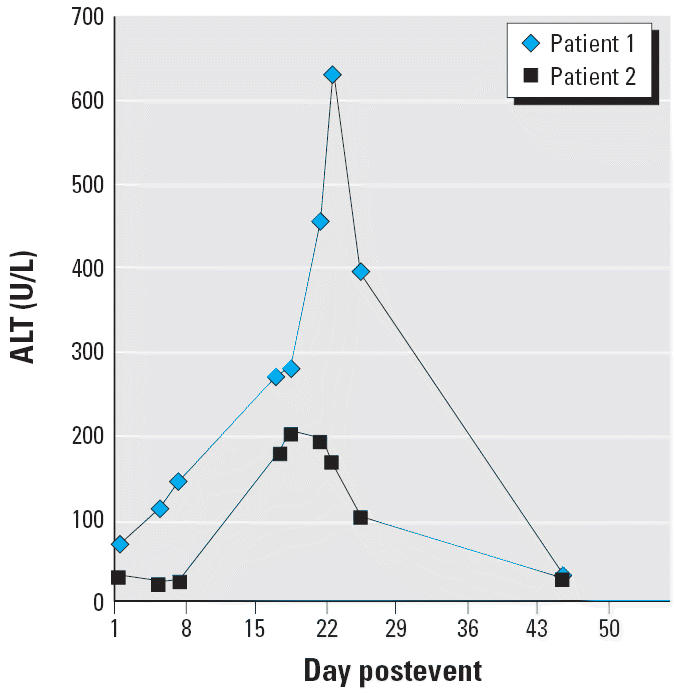
Changes in ALT levels of patients 1 and 2.

He was admitted to the pulmonary ward for observation. Oxygen (6 L/min) was delivered by mask after admission. Exacerbation of his respiratory condition developed on the third day in the hospital. Chest auscultation revealed bilateral basal crackles. Radiography of the chest showed interstitial pulmonary infiltration in both lower lungs. Intravenous hydrocortisone (600 mg/day) and a prophylactic antibiotic (ceftazidime 1 g every 8 hr for 5 days) were administered. His respiratory condition improved after steroid therapy. The steroid dose was decreased gradually and discontinued 13 days after initiation. Elevation of liver enzymes was noted during hospitalization. ALT levels increased to 138 U/L on day 7 and peaked at 625 U/L on day 22 (Figure 1). Serologic investigation was negative for viral hepatitis B and C. No significant abnormal findings were observed on abdominal sonography.

The patient was discharged on the 28th hospital day with a decreasing liver enzyme levels. Two weeks later (6 weeks after the accident), the patient’s liver function returned to normal. In addition, in serial follow-up studies we failed to find any evidence of HIV (human immunodeficiency virus) or autoimmune disease for 18 months.

### Patient 2.

The second patient, a 24-year-old man with an unremarkable medical history, was admitted to our hospital on the same day from the same accident described for patient 1. He had been healthy previously and denied any history of alcohol or drug abuse. He also had dyspnea, cough, chest distress, and a sore throat. Physical examination revealed blood pressure of 120/74 mmHg, heart rate of 84 bpm, and oral temperature of 37.4°C (99.3°F). Arterial blood gas analysis and blood chemistry tests were within the normal ranges. Hematologic tests revealed leukocytosis with neutrophils predominant (Table 1). The leukocytosis returned to normal on the 9th day after the event.

Oxygen was given by face mask after admission. Two days later, the patient experienced progressive respiratory distress. Chest auscultation revealed bilateral expiratory wheezes. A chest radiograph revealed diffuse interstitial infiltrates. High-resolution computed tomography of the chest showed acute inhalation pneumonitis with patchy and small ill-defined nodular areas of ground-glass opacity over all lung fields. Respiratory supports, including steroid therapy and antibiotic prophylaxis were administered as for patient 1. Three days after treatment, his respiratory condition improved and we began to gradually decrease the steroid.

The AST and ALT concentrations increased to a peak of 92 and 195 U/L, respectively, on day 18 (Figure 1). Serologic tests for hepatitis B and C and abdominal sonography showed no abnormal findings. The ALT level decreased to 95 U/L before discharge on the 27th day. Two weeks after discharge, the patient’s liver function returned to normal. In addition, serial follow-up studies failed to find any evidence of HIV or autoimmune disease for 18 months.

## Discussion

HC/ZnO smoke is used by the military to conceal troops, for crowd dispersal, and occasionally in military and civilian firefighting. The acute toxic effects of HC/ZnO smoke on the respiratory tract are primarily attributed to inhalation of hydrated ZnCl_2_ vapor. The vapor is very corrosive and rapidly damages the respiratory mucosal surface (Cullumbine 1957; Greenfield et al. 2002; Hjortso et al. 1988). To the best of our knowledge, the hepatic effects of HC/ZnO are very limited. Pettila et al. (2000) reported three patients with ZnCl_2_ inhalation; all three patients had severe acute respiratory distress syndrome (ARDS). Acute exposure causes the elevation of liver enzymes by day 1 or 2, which peaks from day 18 to day 21 and then returns to normal in 6 weeks (Figure 1). The mechanism of hepatotoxicity of HC/ZnO smoke is still unknown. Several compounds, including ZnO, ZnCl_2_, HC, chlorinated vapors, and medications, may cause hepatic toxicity.

ZnO has not been reported to cause hepatic damage, whereas ZnCl_2_, HC, and chlorinated compounds have great potential to induce hepatotoxicity. Among 12 workers with 4–21 years of exposure to ZnO fumes in the production of brass alloys, no liver disease was reported (ATSDR 2003b; Hamdi 1969). Pettila et al. (2000) reported abnormal liver function (ALT of 119, 131, and 2,570 U/L) in three patients; however, because there was no detailed personal history (e.g., transfusion, alcohol consumption) or viral markers, it was difficult to evaluate the potential hepatotoxicity. Marrs et al. (1988) observed a significant increase in the incidence of fatty liver in mice after repeated exposure to ZnCl_2_ smoke, but the incidence did not increase with the dose, and hepatic toxicity was not observed in the liver of rats and guinea pigs using the same exposure paradigm.

About 5% or less of the compounds in HC/ZnO smoke are released into the air as HC. In one study, Selden et al. (1993) reported that liver function tests were not affected in HC-exposed workers who wore protective clothing. Animal studies have shown that hepatic tissues are moderately vulnerable to HC exposure, especially when exposure occurs by the oral route. Increases in liver weight, increases in serum levels of liver enzymes, centrilobular necrosis, fatty degeneration, hemosiderin-laden macrophages, and hemorrhage were seen in animals exposed to HC [ATSDR 1997; Fowler 1969; Gorzinski 1985; National Toxicology Program (NTP) 1989; Weeks et al. 1979]. In these studies, effects on the liver and kidneys were mild with inhalation exposure and more pronounced with oral exposure. In a study of acute and intermediate-duration inhalation exposure, the only effect noted by Weeks et al. (1979) after 6 weeks of exposure to 260 ppm HC was an increase in liver weight in rats and guinea pigs but not in quail.

About 10% of HC/ZnO smoke is composed of chlorinated compounds (Holmes 1999; Katz et al. 1980; National Research Council 1997). The chlorinated compounds include tetrachloromethane (i.e., carbon tetra chloride, CCl_4_), tetrachloroethylene, and hexachlorobenzene. CCl_4_ has long been known to be a powerful hepatotoxic agent in humans and animals. The principal clinical signs of liver injury in humans who inhale CCl_4_ are a swollen and tender liver, elevated levels of hepatic enzymes in serum, elevated serum bilirubin levels and the appearance of jaundice, and decreased serum levels of proteins such as albumin and fibrinogen (ATSDR 2003a). Liver necrosis was reported in one fatal case involving an alcoholic patient who was exposed to 250 ppm for 15 min (Norwood et al. 1950). High CCl_4_ vapor concentration might have produced liver injury in our patients. However, we could not identify any right upper quadrant tenderness or liver enlargement in either of our patients.

Toxicant- or drug-induced liver injury is a potential complication of nearly every medication (Lee 1995). We needed to rule out the possibility that the observed hepatic toxicity was due to therapeutic drugs. For our cases, we prescribed acetaminophen for fever, steroids, antibiotics, bambuterol, aminophylline, ipratropium, mucolytics for pulmonary symptoms, and a mild sedative for sleeping. The three potential hepatic toxicants are acetaminophen, steroids, and antibiotics; however, acetaminophen taken at recommended doses (0.5–3 g daily) is relatively safe (Norris 2000). One of our cases took < 0.5 mg/day for 2 days, and the other never took it.

Steroid therapy is a standard treatment of ARDS and is applied universally. A thorough search of the literature revealed only a few case reports of suspected corticosteroid-induced hepatomegaly (Nanki et al. 1999). Based on limited data, it is difficult to justify any correlation between corticosteroid use and the hepatitis seen in our patients.

Elevations in liver enzymes and bilirubin have occurred during treatment with ceftazidime. The incidence appears similar to that with other cephalosporins (Meyers 1985). In most cases, liver enzyme elevations have been transient, with levels returning to normal after withdrawal of treatment. Our patients received a 5-day course of ceftazidime for prophylaxis. However, continued deterioration of liver function was observed after ceftazidime was discontinued.

The diagnosis of toxicant-induced liver injury is often obscured by difficulty in determining the precise timing of toxicant ingestion and lack of specific symptoms (Lee 1995; Norris 2000). Central to the diagnosis is a thorough history, including drug exposure and occupational hazards with exposure to chemicals. In addition, the changes of liver enzymes may represent progression on underlying disease, a complication of the underlying disease, or an unrelated episode, such as sepsis or shock (Norris 2000). Confirmation of the diagnosis by a toxicant rechallenge is reliable but rarely justifiable (Lee 1995; Maria et al. 1997; Norris 2000). However, given that these two individuals inhaled the same gas, it is more likely that the effects were due to either the chemicals in the smoke or a combination of the chemicals in the smoke and the drugs, rather than the drugs alone.

The main treatment for toxicant-induced hepatotoxicity is the withdrawal or removal of the agent, supportive care, and alleviation of the symptoms (Lee 1995; Norris 2000). In our cases, there was no treatment beyond what was done for the respiratory effects.

## Conclusion

Inhalation of HC/ZnO smoke may have hepatic effects in addition to the effect of pulmonary distress. The hepatotoxic effect in our patients was attributed to inhalation of high-concentration HC/ZnO smoke in an enclosed area, where several compounds including ZnCl_2_, HC, and chlorinated vapors may have been generated and mixed in the smoke.

## Figures and Tables

**Table 1:** Characteristics and biochemical data of patients 1 and 2 at 4 hr after the event.

	Patient 1	Patient 2	Normal range for males
Age (years)	23	24	
Body weight (kg)	64.5	67	
Body mass index (kg/m^2^)	19	21	18–24
White blood cell (L/mm^3^)	21,900	17,100	4,500–11,000
Neutrophils (%)	93	91	40–70
Lymphocytes (%)	3.8	3.4	19–48
ALT (U/L)	60	24	< 41
AST (U/L)	47	31	< 37
ALP (U/L)	176	123	40–129
GGT (U/L)	61	25	9–40

ALP, alkaline phosphatase
